# Chiral Metal Halide Perovskites: Focus on Lead-Free Materials and Structure-Property Correlations

**DOI:** 10.3390/molecules28166166

**Published:** 2023-08-21

**Authors:** Clarissa Coccia, Marco Moroni, Lorenzo Malavasi

**Affiliations:** Department of Chemistry and INSTM, University of Pavia, Via Taramelli 12, 27100 Pavia, Italy; clarissa.coccia01@universitadipavia.it (C.C.); marco.moroni@unipv.it (M.M.)

**Keywords:** metal halide perovskites, chiral materials, optical properties, structure of solids

## Abstract

Hybrid organic–inorganic perovskites (HOIPs) are promising materials in several fields related to electronics, offering long carrier-diffusion lengths, high absorption coefficients, tunable band gaps, and long spin lifetimes. Recently, chiral perovskites have attracted huge interest thanks to the possibility of further widening the applications of HOIPs. Chiral materials, being intrinsically non-centrosymmetric, display several attractive physicochemical properties, including circular dichroism, circularly polarized photoluminescence, nonlinear optics, ferroelectricity, and spin-related effects. Recent studies have shown that chirality can be transferred from the chiral organic ligands into the inorganic perovskite framework, resulting in materials combining the advantages of both chirality and perovskite superior optoelectronic characteristics. As for HOIPs for photovoltaics, strong interest is currently devoted towards the development of lead-free chiral perovskites to overcome any toxicity issue. While considering the basic and general features of chiral HOIPs, this review mainly focuses on lead-free materials. It highlights the first attempts to understand the correlation between the crystal structure characteristics and the chirality-induced functional properties in lead and lead-free chiral perovskites.

## 1. Introduction

### 1.1. General Overview

In recent years, hybrid organic-inorganic perovskites (HOIPs) have emerged as promising materials for solar cells, light-emitting diodes, photodetectors, and devices for optoelectronics and energy harvesting [1,2,3,4,5,6], thanks to the outstanding quantum yields and easy emission tunability. Further expansion of these applications may be achieved through chirality, i.e., the property of an object not being superimposable to its mirror image [7]. In this context, HOIPs can be imparted with chiroptical properties by the employment of chiral organic cations, generating compounds that combine the advantages of both the chiral molecules and the halide perovskites. Indeed, while the asymmetric ligand induces nonlinear optical (NLO) properties in the final materials, the HOIPs skeleton shifts the asymmetric absorption and emission maxima in the UV-Vis-IR region, through a mechanism known as Chirality Transfer Mechanism, which is currently being investigated [8]. Although a pioneering work on chiral perovskites was carried out in 2003 [9], characterizing the crystal structure of a 1D HOIP, the first 2D HOIP appeared in 2006 [10]. The chiroptical properties of these materials started being deeply investigated only in 2017 [11], with the first characterization of the dichroic behaviour of various HOIPs through circular dichroism (CD). Immediately after, the field started growing significantly, as the chemical and structural tuning of HOIPs offered a unique playground for developing novel and more efficient materials featuring higher spin selectivity, superior circular dichroism, improved stability, and so on. Although the field is still at an early stage, it is clear that a number of functional properties, such as CD, Rashba-Dresselhaus (RD) spin splitting [12], conductivity, ferroelectricity or piezoelectricity, strictly depends on the chemical nature, stoichiometry, network dimensionality (0D, 1D, or 2D), hydrogen bonding, and other structural parameters, such as the octahedral distortions.

For this reason, a deep and reliable investigation of the crystal structure is fundamental to understand the theoretical development of novel materials. With this concept in mind, in this review, we selected a series (mostly lead-free) of chiral perovskites where crystal structures were solved and reported. In this respect, while reporting the corresponding functional properties observed, we also focus on the attempts to correlate the structural properties with the chiroptical features.

### 1.2. Chirality and Related Functional Properties

As anticipated, an object can be defined as chiral if it is not superimposable to its specular counterpart. To achieve this condition, it must not contain symmetry elements of the second kind, such as inversion centers, mirror planes or rotation-reflection axes, i.e., it must crystallize in one of the 65 space groups known as Sohncke groups. More details on chirality in the solid state can be found in ref. [13]. From a functional viewpoint, a chiral crystal can exhibit several intriguing photophysical properties upon irradiation with a polarized source. First, it can display CD and circularly polarized photoluminescence (CPL), associated with the difference in absorption and emission intensity, respectively, of the left-handed and right-handed radiation. This difference can be quantified by the asymmetry factors. In the case of CD, it can be calculated as

$$g=\frac{A_{L}-A_{R}}{A_{L}+A_{R}}$$

where *A_L_* and *A_R_* represent the left-handed and right-handed intensities of the absorbed radiation. As for CPL, it is defined as

$$g=\frac{I_{L}-I_{R}}{I_{L}+I_{R}}$$

where *I_L_* and *I_R_* stand for the left-handed and right-handed emitted intensities. It has to be stressed that the asymmetry factor is the parameter that should always be reported when dealing with chiral perovskites since the ellipticity (*θ*) itself is not a normalized value. Then, a chiral HOIP can feature a second harmonic generation (SHG) response, a phenomenon associated with the generation of a photon with twice the energy of the two incident ones upon the annihilation of these two. SHG active materials are extremely useful in different photonics applications, such as frequency upconversion and laser modulation [14]. 

From a microscopic point of view, the band structure and spin states are perturbed in non-centrosymmetric materials, leading to effects such as the chirality-induced spin selectivity (CISS), affecting several electron processes, namely electron transmission, electron transport and chemical reactions, or the so-called Rashba-Dresselhaus spin (RD) splitting. The latter is caused by the inversion symmetry breaking due to the presence of a spin-orbit field, which causes a lift in the energy of two otherwise degenerate electronic bands.

Moreover, ferroelectricity and piezoelectricity are interesting functional properties in non-centrosymmetric crystals, promising for a few practical applications. Ferroelectricity, i.e., the generation of a spontaneous electric polarization that can be reversed upon applying an external electric field, can be utilized to construct capacitors, sensors, memories, and other devices [15]. Similarly, piezoelectricity, referring to the property of materials undergoing physical deformations upon applying an electric field or, inversely, to produce an electrical charge upon mechanical deformation, is promising for building up devices for energy harvesting applications [16].

## 2. Chiral Perovskites

**Figure 1:**
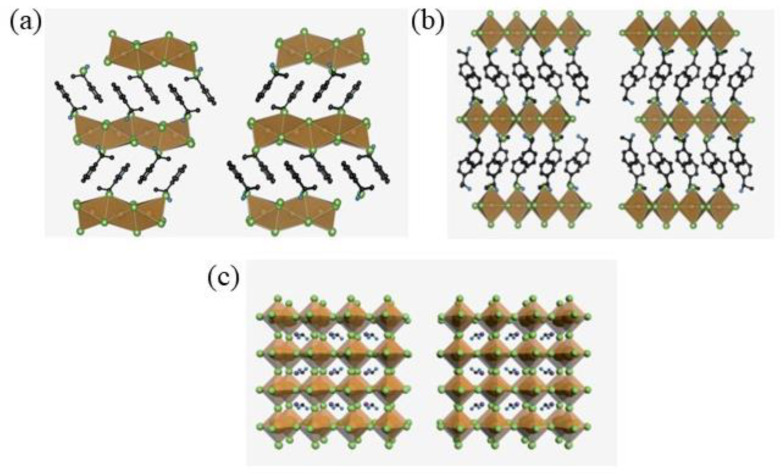
Structure of Chiral Perovskites: (**a**) 1D structure; (**b**) 2D structure; (**c**) 3D structure. Reprinted with permission from Ref. [4]. 2021, Wiley.

The general chemical formula for 3D (Figure 1a) HOIPs is AMX_3_, where A represents a monovalent cation, M is a metallic bivalent cation, and X is a halogen anion. From a structural point of view, 3D perovskites display corner-shared [MX_6_] octahedrons, which form a 3D framework with the A cations located in the framework cavities. For 3D perovskites, theoretical calculations revealed that the generation of stable compounds requires that the A cation is small enough to fit within the inorganic framework cavities. Due to this constraint, 3D chiral HOIPs remain in the theoretical development stage, as their instability hinders their practical optoelectronic applicability. To overcome these problems, 2D chiral HOIPs have been introduced and developed (Figure 1b). Compared to the 3D counterparts, these can be regarded as a horizontal slicing of the 3D frameworks with the incorporation of a bigger organic cation. 2D HOIPs exhibit the generic chemical formula of (R-NH_3_)_2_A*_n_*_−1_MX_3*n*+1_, where *n* denotes the number of inorganic layers between two layers of organic cations, and R-NH_3_ represents the chiral ligand inserted between two inorganic layers. At the state of the art, this family of chiral HOIPs is the most widely investigated since the optical and physical properties can easily be tuned by changing *n* and chemical composition.

In contrast to 2D chiral HOIPs, studies on 1D and 0D HOIPs are scarce [1]. 1D chiral HOIPs (Figure 1c), featuring the generic chemical formula of R-NH_3_MX_3_, can be created when chiral organic cations and metallic cations are present in a 1:1 ratio. 0D HOIPs, instead, can be produced when the geometric sizes of 3D, 2D and 1D chiral HOIPs shrink to a few nanometres. These low-dimensional structures are more correctly defined as perovskite derivatives of perovskite-hybrid halides since, in general, they do not feature any corner sharing network of the octahedra. Here, we will give an overview of the different chemical structures of chiral perovskites, discussing the synthetic strategies and the chiroptical properties.

### 2.1. 0D Chiral Perovskites

**Figure 2:**
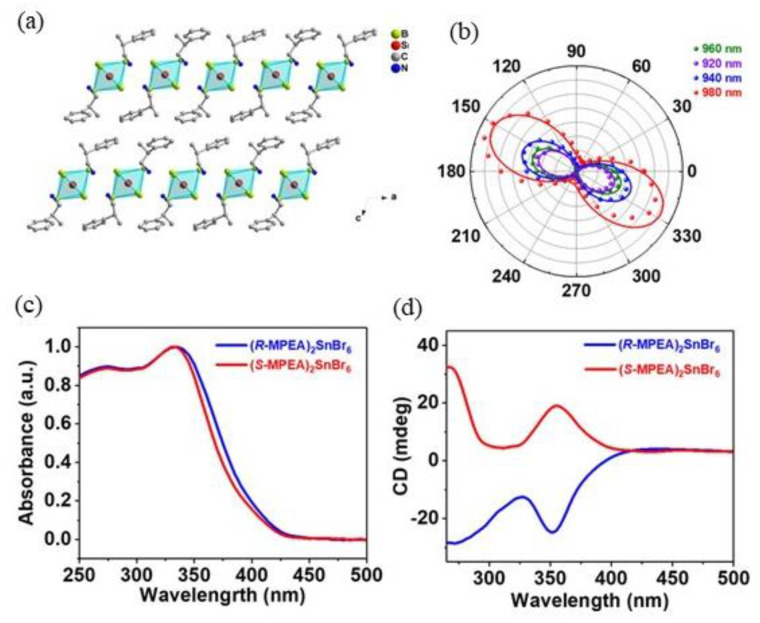
(**a**) Structure of (*R*-MPEA)_2_SnBr_6_; (**b**) SHG measurement; (**c**) UV-Vis and (**d**) CD spectra. Reprinted with permission from Ref. [17]. 2021, Wiley.

In 2021 Zhao et al. synthesized an environmentally friendly zero-dimensional (0D) lead-free chiral perovskite to evaluate the non-linear optical properties, such as second harmonic generation (SHG), and to overcome the toxicity and instability of perovskites containing Pb^2+^ [17]. They introduced the chiral amine ß-methylphenethylamine (MPEA) into the perovskites structure by solution method, crystallizing (*R/S*-MPEA)_2_SnBr_6_ and (*rac*-MPEA)_2_SnBr_6_ starting from the *R/S/rac*-MPEABr and SnCl_2_ • 2H_2_O in a 2:1 molar ratio, dissolving these reactants in HBr. This solution was left open, and after 24 h, plate-like crystals were obtained. Thanks to Single Crystal X-ray diffraction (SC-XRD), the structure of these compounds was resolved and is reported in Figure 2a. Both enantiomers adopt the chiral space group *P*2_1_, while the racemic material is in the *P*2_1_/*c* space group. The authors optimized the host-guest engineering of these 0D mononuclear molecules and obtained efficient second-order NLO properties thanks to the incorporation of the chiral cations. They focused on UV-Vis absorption and NLO properties such as CD, reported in Figure 2c, in which it is possible to observe an optical band gap around 2.69 eV for all of the three compounds and a strong CD response at 352 nm, opposite for the two enantiomers.

Moreover, the removal of intrinsic centrosymmetry by the presence of the chiral amine led to a strong SHG response for the compounds crystallizing in the *P*2_1_ space group (Figure 2b). Indeed, this chiral material reports an SHG signal band when the excitation laser tunes its wavelength between 800 nm and 1200 nm, leading to a high polarization ratio. They also concluded that this strong NLO effect has a two-photon nature, and the optical stability is higher than those typically reported for perovskite materials, as determined by considering the high value of laser-induced damage threshold (LDT) [17].

**Figure 3:**
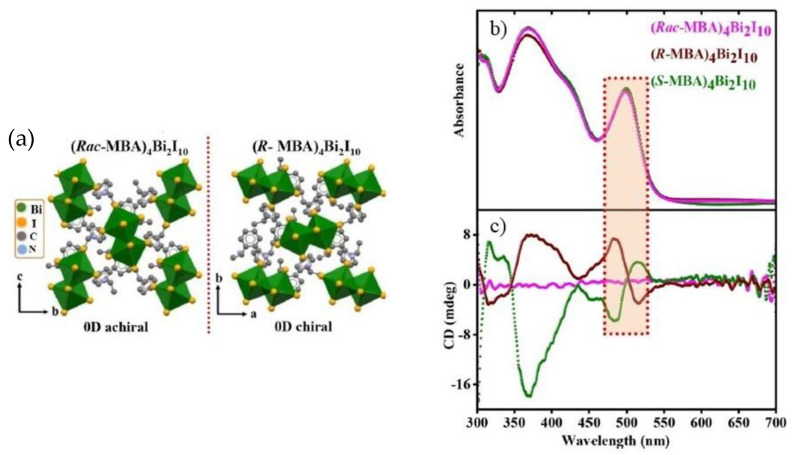
(**a**) Structure of (*R/rac*-MBA)_4_Bi_2_I_10_; (**b**) UV-Vis spectra; (**c**) CD spectra. Reprinted with permission from Ref. [18]. 2022, American Chemical Society.

In 2022 Rajput et al. proposed another lead-free zero-dimensional system based on bismuth, preparing (*R/S/rac*-MBA)_4_Bi_2_I_10_ by solution method [18]. After dissolving bismuth (III) oxide in hydroiodic and hypophosphorous acid at 100 °C, they added *R*- *S-. rac*- methylbenzylamine (MBA) to the solution and cooled at room temperature at 5 °C per hour. The obtained crystals were characterized by SC-XRD (Figure 3a), observing that all the samples crystallize in the monoclinic system with a 0D structure, with *P*2_1_ space group for the two enantiomers and *P*2_1_/*c* for the racemic. Noteworthy, the presence of chirality in the two enantiomers is confirmed by the absence of the rotation axes or inversion centres, granted by the crystallization in the Sohncke space group. The effect of chirality on the optical properties was evaluated by conducting UV-Vis absorption and CD measurements on thin films of the three samples obtained by spin coating. From the UV-Vis spectrum (Figure 3b), a sharp band around 500 nm can be appreciated, which leads to a value of exciton binding energy of 150 meV. This result is quite significant in terms of NLO properties, as it indicates the confinement of the electron-hole pair in the inorganic part of the structure. The CD spectra shown in Figure 3c report a bipolar signal around 500 nm, as expected, probably due to the spin-orbital coupling.

The authors further investigated the photophysical excitonic properties by means of temperature-dependent photoluminescence (PL) measurements. The PL spectra, instead, show a peak around 512 nm until 100 K, after which the peak moves to higher wavelength values. The lowest temperature investigated, namely 7 K, displays the highest peak intensity due to the extinction of the non-radiative decay. Interestingly, the observed excitonic emission has not been seen in other chiral Pb-free perovskites, and it is probably linked to fewer defects in the structure inhibiting the nonradiative decay. Moreover, the authors observed small Stokes shifts and relatively small lifetimes, suggesting that the PL emission at 550 nm involves shallow defects close to band edges [18].

**Figure 4:**
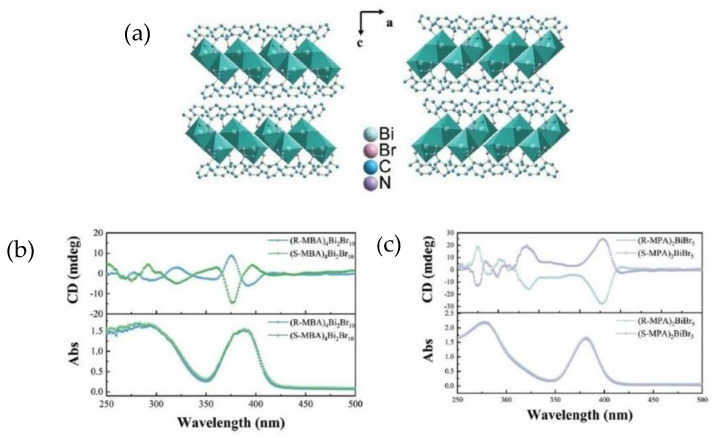
(**a**) Structure of (*R/S*-MBA)_4_Bi_2_Br_10_; (**b**) UV-Vis and CD spectra of (*R/S*-MBA)_4_Bi_2_Br_10_; (**c**) UV-Vis and CD spectra of *R/S*-MPA)_2_BiBr_5_. Reprinted with permission from Ref. [19]. 2023, Wiley.

In 2023 Jiang et al. proposed a comparison between two Bi-based chiral perovskites, displaying different organic cations and dimensionality [19]. Indeed, while one is 0D and features the generic molecular formula (*R/S*-MBA)_4_Bi_2_Br_10_, (MBA = α-phenylethylenamine), the other is 1D and displays the general formula (*R/S*-MPA)_2_BiBr_5_ (MPA = 1-phenylpropane-amine). The two organic cations only differ in the length of the chain, as MPA possesses 1 C atom more, and the authors try to enlighten the role of ligand nature on the chiroptical properties. Both perovskites have been synthesized by the solution method, followed by a cool-down process to obtain the single crystals, which were characterized through SC-XRD. The crystal structure, reported in Figure 4a, was solved for all the compounds, unveiling that (*R/S*-MBA)_4_Bi_2_Br_10_ crystallizes in the monoclinic *P*2_1_ space group while (*R/S*-MPA)_2_BiBr_5_ in the orthorhombic *P*2_1_2_1_2_1_ one. The latter shows a higher rigidity and a stronger asymmetry due to the confinement created by the 1D inorganic chains that induced the amines to arrange in a neater way. The authors performed UV-Vis absorption and CD measurements, reported in Figure 4b and c, respectively. The UV-Vis spectra of the zero-dimensional perovskites show two peaks at 286 nm and 386 nm, while the CD spectra exhibit symmetric curves and the Cotton effect. UV-Vis measurements performed on the 1D perovskites show two peaks at higher wavelengths vs. the 0D system, while CD measurements also indicated, in this case, opposite profiles characterized by the Cotton effect after the excitation absorption peak. An interesting comparison can be done on the *g*_CD_ values, higher in the case of (*R/S*-MPA)_2_BiBr_5_ and easily explainable by the connection between the anisotropic coefficient and the magnetic dipole moment, related to the disposition of the octahedra in the two cases (see Section 3). Another important characterization on which the authors focused was the SHG response, observed in both (*R/S*-MPA)_2_BiBr_5_ and (*R/S*-MBA)_4_Bi_2_Br_10_ with different magnitudes. Indeed, (*R/S*-MPA)_2_BiBr_5_ exhibits a SHG response four times higher than (*R/S*-MBA)_4_Bi_2_Br_10_, again due to the arrangement of the structure, as will be explained in Section 3 [19]. 

### 2.2. 1D Chiral Perovskites

**Figure 5:**
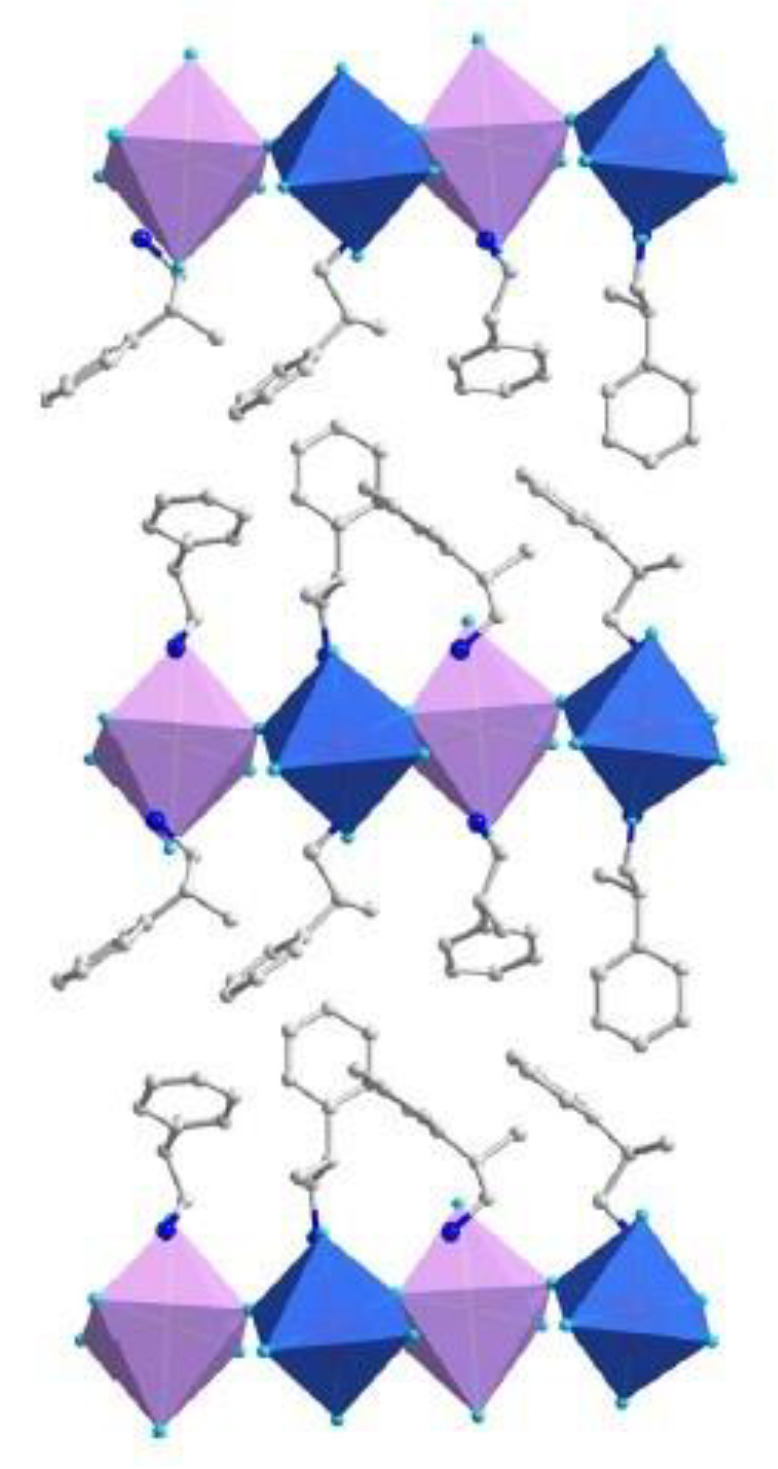
Structure of [R-β-MPA]_4_AgBiI_8_ where BiI_6_ octahedra, purple; AgI_6_ octahedra, blue. Reprinted with permission from Ref. [20]. 2021, Wiley.

In 2021 Li et al. synthesized a lead-free, one-dimensional double chiral perovskite with the chemical formula [(*R/S*)-β-MPA]_4_AgBiI_8_ (MPA = methylphenethylammonium), conducting CD and CPL measurements and underlining the performances of double hybrid perovskites and the possibility to work without using lead [20]. They prepared the samples by solution method, using AgO_2_ and Bi_2_O_3_ as metal precursors in hydroiodic acid and adding MPA (*R* or *S*) after the dissolution of the precursors. The authors solved the structure by SC-XRD (Figure 5), disclosing that both enantiomers crystallize in the *P*2_1_ space group and feature corner-sharing BiI_6_ and AgI_6_ octahedra layers, stacking along the *c* axis, where the chiral β-MPA cations are distributed. 

**Figure 6:**
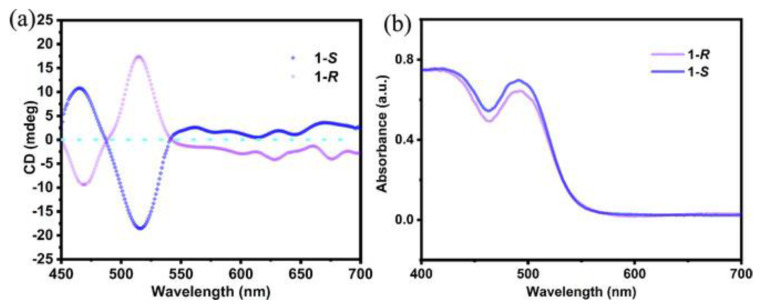
(**a**) CD spectra; (**b**) UV-Vis spectra. Reprinted with permission from Ref. [20]. 2021, Wiley.

To prove the chirality transfer, they performed CD measurements, in which the CD signals are located at the characteristic absorption wavelength of the chiral double perovskite (Figure 6a,b), proving the direct chirality transfer from the organic cation to the inorganic framework. To investigate the chiroptical properties, they conducted CPL measurements, allowing for the determination of the anisotropic factor related to the photocurrent, calculated through the simple equation:

$$g_{Iph}=\frac{I_{R}-I_{L}}{I_{R}+I_{L}}$$

For the *R* enantiomer *g*_Iph_ turns out to be 0.22, an interesting result for a double lead-free perovskite. This high value can be correlated to the spin-orbit coupling induced by the heavy elements Bi and I, which allow the Rashba splitting in the chiral structure [20].

**Figure 7:**
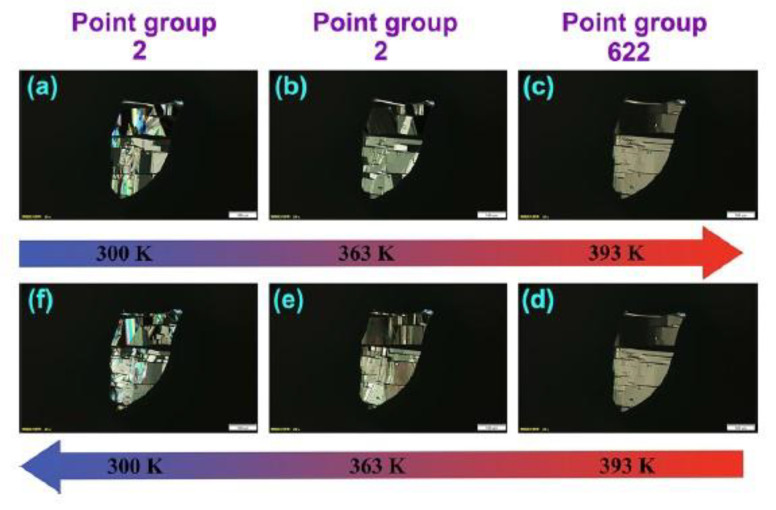
(**a**–**f**) Evolution of ferroelastic domains under the variation of temperature for [*R*-EQ]PbI_3_ with the scale bar of 100 μm. Reprinted with permission from Ref. [21]. 2022, American Chemical Society.

While no other lead-free 1D chiral perovskites have been reported to date, it is worth highlighting the important results obtained on a couple of Pb-containing materials which may be used as starting points to further expand the family of lead-free systems. In 2022 Fu et al. synthesized the ferroelastic lead-iodide perovskite [EQ]PbI_3_ [21], where EQ stands for N-ethyl-quinuclidine, introducing, after the synthesis, a hydroxyl group in position 3 of the aminic quinuclidine ring to convert the molecule into a chiral one and generating a couple of enantiomers_._ The *R*-EQ sample undergoes a paraelectric/paraelastic-ferroelectric/ferroelastic phase transition, which is the focus of this paper. Indeed, the sample presents a phase transition at 355 K and passes from the chiral non-centrosymmetric space group *P*2_1_ to the *P*6_3_22 one, turning from ferroelectric into paraelectric. This transition is confirmed by SHG measurements and by polarized light microscopy measurements, the latter allowing to study the evolution of the ferroelectric domains. The results reported in Figure 7 showed that the ferroelectric domains diminish, increasing the temperature, in agreement with the phase transition from the chiral space group to the symmetric one [21].

In 2023 Liu et al. prepared two different pairs of one-dimensional chiral perovskites, one only containing a single chiral cation and cited as mono-cation, while the second containing two different cations, one of them achiral and labelled as mixed-cation [22]. The aim was to investigate the influence of an achiral molecule in affecting or modulating the chiroptical properties. The first one, named (*R/S*-AMP)_2_Pb_3_Br_10_ (AMP = *R/S*-2-aminomethylpyrrolidine), has been synthesized by solution method starting from hydrobromic acid, lead (II) acetate and the amine. The second one, *R/S*-AMP(DMA)PbBr_5_ (DMA = dimethylammonium), was also obtained by solution method but adding the mixture of organic linkers instead of the single one. As determined by SC-XRD, all four compounds crystallize in a *P*2_1_2_1_2_1_ space group. XRD also underlined that the interchain spacing is lower in the case of the mixed-cation samples, providing stronger interactions between the achiral blocks and the chiral molecules. By means of UV-Vis measurements, the value of the band gaps, i.e., 3.43 eV for (*R/S*-AMP)_2_Pb_3_Br_10_ and 3.38 eV for *R/S*-AMP(DMA)PbBr_5_ were determined, while PL spectra showed two peaks for the mono-cation samples, one at 440 nm and the other at 520 nm, and one broad peak at 550 nm for the mixed-cation. The authors calculated the PL quantum yields and observed higher values for the mono-cation perovskite. This observation can be linked to the structure of these samples, as the mixed-cation perovskite presents a higher distortion of the octahedra, and the *D* (distortion index) parameter is reversely correlated to the PLQY. The CD spectra unveiled in both cases, i.e., (*R/S*-AMP)_2_Pb_3_Br_10_ and *R/S*-AMP(DMA)PbBr_5_, the presence of opposite signals, which prove the symmetry breaking in each sample. However, the second couple (mixed-cation samples) produced a CD signal twice higher than the first couple, associable with a larger magnetic transition dipole moment induced by the insertion of the achiral cations. In terms of CPL measurements, the anisotropic coefficient (*g*_lum_) is higher in the mixed-cation samples. The authors ascribed this result to the stronger hydrogen bond interactions in the supramolecular network, which lead to a more effective chirality transfer which amplifies the structure chirality [22]. 

### 2.3. 2D Chiral Perovskites

**Figure 8:**
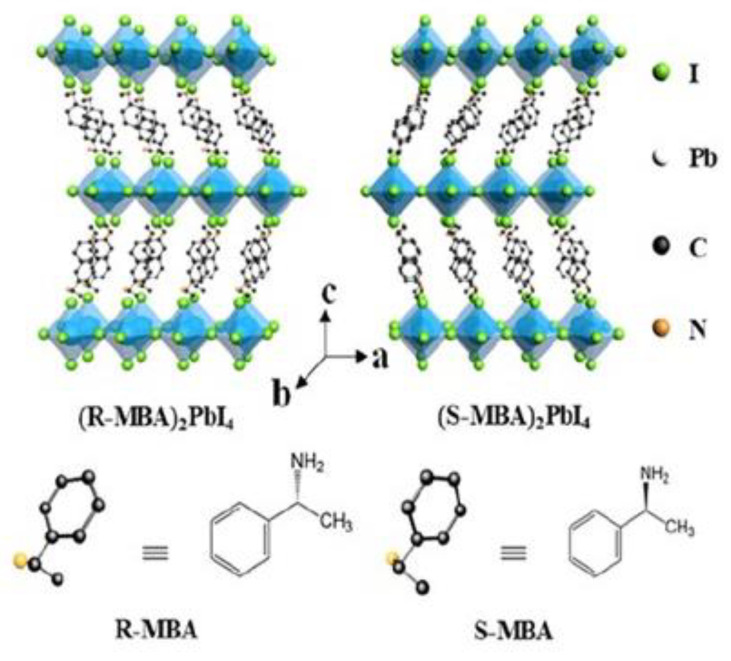
Structure of (*R/S*-MBA)_2_PbI_4_. Reprinted with permission from Ref. [23]. 2019, American Chemical Society.

In 2019 Ma et al. proposed one of the first works centred on chiral two-dimensional perovskites [23]. They synthesised (*R/S*-MBA)_2_PbI_4,_ where MBA stands for C_6_H_2_C_2_H_4_NH_3,_ working in solution and obtaining single crystals, which were analysed by SC-XRD. They found a structure composed of a single corner-sharing octahedral layer between two layers of chiral organic chains (Figure 8). The enantiomers crystallize in the *P*2_1_2_1_2_1_ space group while the racemic in the *P*2_1_/*a* one.

**Figure 9:**
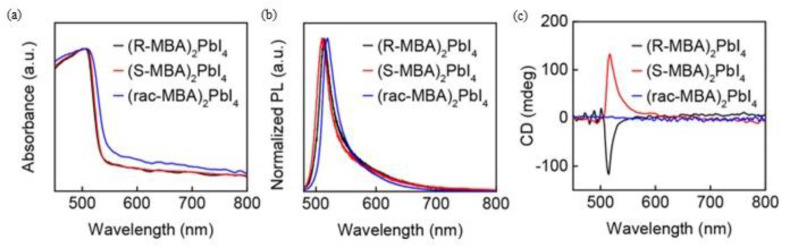
Normalized absorption (**a**) and steady-state PL spectra (**b**) of (*R*-MBA)_2_PbI_4_, (*S*-MBA)_2_PbI_4_, and (*rac*-MBA)_2_PbI_4_ microplates obtained by mechanical exfoliation. (**c**) CD spectra of (*R*-, *S*-, and *rac*-MBA)_2_PbI_4_ films. Reprinted with permission from Ref. [23]. 2019, American Chemical Society.

The authors focused their attention on the optical properties performing CD and PL measurements. The UV-Vis spectra, reported in Figure 9a, show absorption edges at 533 nm for the *R/S* samples and 555 nm for the racemic one. The little shift can be linked to differences in the morphology of the samples. The CD measurements (Figure 9b) highlight opposite peaks for the two enantiomers and a flat signal for the racemic, confirming the incorporation of the chiral cation. Steady-state PL, reported in Figure 9c, showed an emission peak probably originating from a free exciton emission, while CPL allowed once again to confirm the correct insertion of the chiral cation and the chirality transfer to the inorganic framework. At this point, they quantified the degree of circularly polarized PL working at different temperatures, noticing that the degree of polarization tends to decrease with the increase in temperature. This phenomenon is probably due to the lattice distortion, decreasing with the temperature increase and leading to a chirality reduction [23].

**Figure 10:**
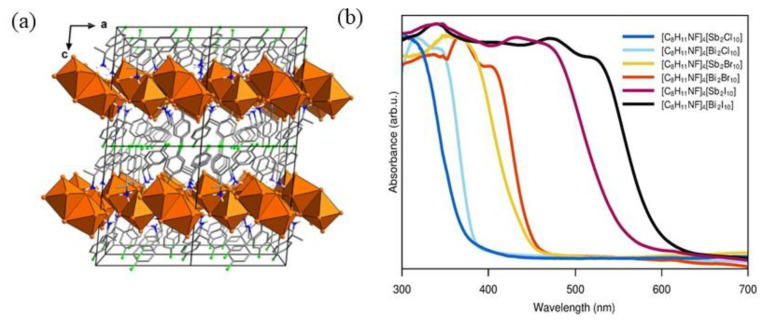
(**a**) Structure of [(R)-1-(4-F)PEA]_4_[Sb_2_Cl_10_]; (**b**) UV-Vis spectra of all compounds. Reprinted with permission from Ref. [24]. 2020, American Chemical Society.

After this first example of Pb-based 2D chiral perovskite, since 2020, the attention quickly shifted towards lead-free materials, aiming to overcome the lead toxicity issues. Dehnhardt et al. obtained an isomorphous family of organic-inorganic metal halide materials with general formula [(*R*)-1-(4F)PEA]_4_[E_2_X_10_] [24], where PEA is phenylethyleammonium, E is Sb or Bi, and X stands for Cl, I and Br. The choice of Sb and Bi was undertaken to overcome both the Pb toxicity and the Sn instability. The authors synthesized six compounds by solution method, dissolving Sb_2_O_3_ and Bi_2_O_3_ in HX acid, adding the amine, heating to reflux, and then cooling down to room temperature. The crystals analysed by SC-XRD (Figure 10a) crystallize in the *P*2_1_ space group, underlying the possibility of nonlinear optical properties. In Figure 10a, the crystal structure of [(*R*)-1-(4-F)PEA]_4_[Sb_2_Cl_10_] is reported as a representative example. The structure presents two layers, one inorganic and the other composed of organic molecules. The authors performed UV-Vis measurements (Figure 10b), observing a redshift passing from Cl to Br and I as expected in HOIPs. By changing the E cations, they did not observe significant shifts, except in the iodide compounds where a visible redshift can be appreciated passing from Sb to Bi. The authors then conducted SHG experiments, observing the absence of SHG response in the iodide compounds, ascribable to the inhomogeneity of the crystals or the instability of the samples under the laser [24].

**Figure 11:**
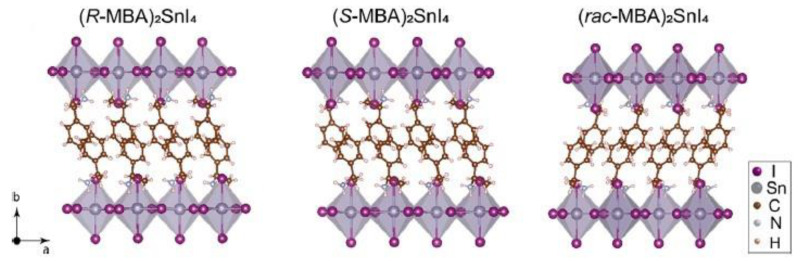
Structure of compounds (*R/S/rac*-MBA)_2_SnI_4_ [25]. Reprinted with permission from Ref. [25]. 2020, American Chemical Society.

In the same year, Lu et al. synthesized chiral HOIPs based on Sn, starting from the synthesis of the three compounds (*R/S/rac*-MBA)_2_SnI_4_ (MBA = methylbenzylamine) and preparing the Sn/Pb alloyed samples (*R*-MBA)_2_Pb_1−X_Sn_x_I_4_ [25] to tune the optical properties. The synthesis of the pure Sn compounds was made by the solution method, resulting in rod-like crystals characterized by SC-XRD. The *R*- and *S*- enantiomers crystallize in the *P*2_1_2_1_2_1_ space group, while the racemic one into the *Pnma* space group (Figure 11).

**Figure 12:**
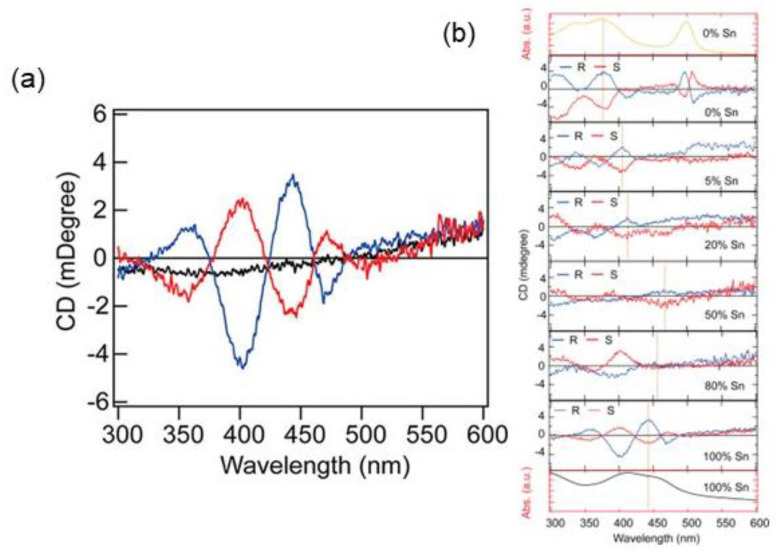
(**a**) CD spectra of (*R/S/rac*-MBA)_2_SnI_4_; (**b**) CD spectra of (*R/S/rac*-MBA)_2_Pb_1−X_Sn_x_I_4_. Reprinted with permission from Ref. [25]. 2020, American Chemical Society.

The NLO properties of the Sn compounds, characterized by CD (Figure 12a), showed distinct signals for the enantiomers. However, the peaks are characterized by a broad profile, possibly because of structural distortion. The CD signal variations upon Pb introduction are reported in Figure 12b, evidencing peak shifts across all wavelength ranges and demonstrating that it is possible to modulate the chiro optical properties by tuning the electronic structures of 2D chiral HOIPs [25].

**Figure 13:**
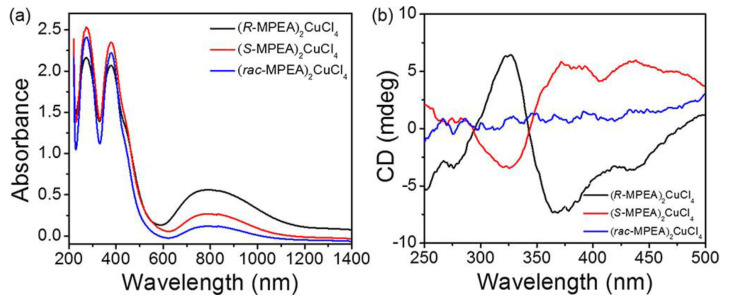
UV–vis–NIR absorption spectra (**a**) and CD spectra (**b**) of (*R*-MPEA)_2_CuCl_4_, (*S*-MPEA)_2_CuCl_4_, and (*rac*-MPEA)_2_CuCl_4_. Reprinted with permission from Ref. [26]. 2020, American Chemical Society.

Other lead-free perovskites were reported by Sun et al. in 2020, proposing the synthesis of (*R/S*-MPEA)_2_CuCl_4_ and investigating CD and ferromagnetic behaviour [26]. First, the authors synthesized both enantiomers by solution method and retrieved the crystal structure through SCXRD. Then, they investigated the optical and NLO properties, such as UV-Vis-NIR and CD. The authors enlightened some important qualities of 2D chiral perovskites, such as the possibility of achieving spin-polarized PL without an external magnetic field, which encouraged the exploitation of chiral ferromagnetic perovskites [27]. The UV-Vis spectra (Figure 13a) present two peaks, one around 397 nm ascribed to an excitonic feature and the other at 274 nm associated with the π transition of the organic cation. Figure 13b reports the CD spectra, showing a strong opposite signal for the enantiomers and a flat signal for the racemic. In terms of magnetic properties, the authors reported hysteresis loops, confirming that these materials report spontaneous magnetization with a saturation value of up to 12.5 emu g^−1^.

Moreover, the authors investigated the magneto-chiral dichroism (MChD), a magneto-optical effect where the absorption coefficient of the chiral compounds for an unpolarized light beam depends on how the magnetic field is applied, i.e., parallel or antiparallel to the propagation direction of the light beam. MChD signals with opposite signs in their optical response were observed for the two enantiomers, resulting in perfect mirror images. Moreover, the different peaks were associated with the electronic structure of the chiral HOIPs [26].

**Figure 14:**
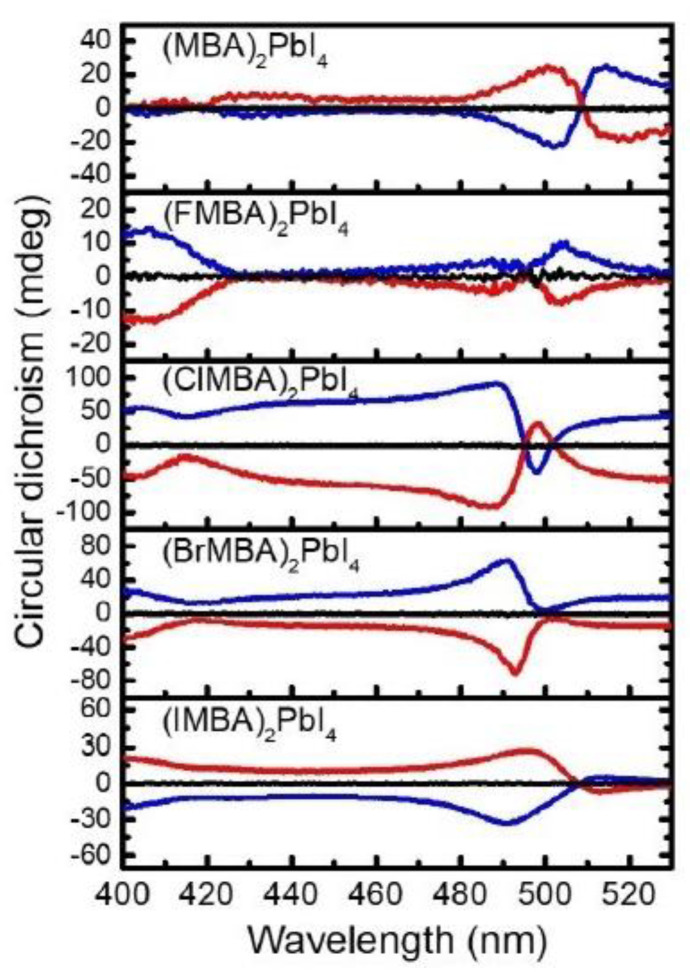
CD spectra of the *R*- (blue), *S*- (red), and *rac*- (black) fabricated HOIPs films including (MBA)_2_PbI_4_, (FMBA)_2_PbI_4_, (ClMBA)_2_PbI_4_ (BrMBA)_2_PbI_4_, and (IMBA)_2_PbI_4_ series. Reprinted with permission from Ref. [28]. 2021, Wiley.

In terms of analogous lead-based perovskites, Lin et al. in 2021 studied the CD and CPL properties of 2D HOIPs with methylbenzilamine (MBA) as organic cation and with different halogen atoms as substituents at the para position of the phenyl group [28]. All the derivatives, namely (XMBA)_2_PbI_4_ (X = H, F, Cl, Br, I), were evaluated through powder XRD regarding the *d*-spacing of the HOIP films. The authors underlined how this value enhances going from no substitution to F, Cl, Br, and I, proving the correct incorporation of the proper cation. The optical properties were studied by UV-Vis absorption and PL measurements on thin films. All the compounds presented a sharp absorption peak at 497 nm and an emissive one at 515 nm, demonstrating that this optical property is independent of the presence and nature of the halogen substituent. On the other hand, the CD spectra (Figure 14) exhibit the strongest intensity for (ClMBA)_2_PbI_4_, possibly because of the halogen-halogen interaction in the substituted system, which improves the rotational strength. Based on this investigation, the authors reported that optimal angular momentum and *d*-spacing are key features to optimize the chiro optical properties of the final materials [28]. 

## 3. Structure-Property Correlations

As anticipated in Section 1, it is well established that the chiroptical features of HOIPs and the chirality transfer mechanism are strictly related to their structural characteristics. However, unique and reliable structural parameters governing the chiroptical properties are still missing, prompting the research to put efforts into this perspective. So far, several correlation attempts have been reported in the literature, which are detailed in the following of this section. Noteworthy, most of them were carried out on Pb-based perovskites, thanks to the higher number of available phases with solved crystal structures, emphasizing the need for the investigation of these aspects also for lead-free systems. The structural parameters proposed so far are mainly related to the distortion of the metal-halide octahedra. For example, the distortion index (*D*) and the octahedral elongation (λ_oct_) are referred to as the metal-halogen bond length, while the octahedral angle variance (σ^2^) is calculated on the *cis* halogen-metal-halogen angles. Moreover, another parameter related to the octahedral bond angles, labelled as Δ*β*, has been proposed in a recent work [29].

Concerning 0D chiral perovskites, in 2022, Rajput et al. attempted to correlate for the first time the bond length distortion index (*D*) and bond angle variance (σ^2^) with the extent of chirality, investigating the (*R/S*-MBA)_4_Bi_2_I_10_ compounds as well as other bismuth-iodide 0D structures reported in the literature [24,30,31,32]. The results were defined by themselves as not satisfactory, as they found out that these parameters are not related to the chirality of the system. However, they disclosed that the structures displaying isolated [BiI_6_]^3−^ octahedra display substantially smaller *D* and σ^2^ values compared to the samples featuring edge-shared or face-shared ([Bi_2_I_10_]^4−^ or [Bi_2_I_9_]^3−^) octahedra, the latter displaying the highest *D* value.

In 2023 Jiang et al. investigated (*R/S*-MBA)_4_Bi_2_Br_10_ and (*R/S*-MPA)_2_BiBr_5_, correlating the information resulting from CD and SHG with the crystal structure features. They disclosed a higher *g*_CD_ in (*R/S*-MPA)_2_BiBr_5_ vs. (*R/S*-MBA)_4_Bi_2_Br_10_ and associated it with the proximity of the octahedra. Indeed, in the 1D perovskite, the [BiBr_6_]^2-^ octahedra are stacked together, while in the 0D one, the [Bi_2_Br_10_]^4−^ octahedra are far from each other. Closer octahedra in the structure lead to a higher influence on the magnetic dipole moment, thus resulting in a higher value of *g*_CD_. Another difference comes from the SHG response, which is four times higher in the case of (*R/S*-MPA)_2_BiBr_5_. In this circumstance, its higher value was associated with the greater rigidity of the 1D system and the infinite extension of the chains along *an* axis. This led to a more regular arrangement of the organic amines, thus favouring a more significant symmetry breaking. 

As for the 1D HOIPs, a structure-photoluminescence (PL) properties correlation was reported for the first time in 2023 by Liu [22], working on (*R/S*-AMP)_2_Pb_3_Br_10_ and *R/S*-AMP(DMA)PbBr_5_ (see above), correlating *D*, σ^2^ and the octahedral elongation (λ_oct_) with the PL properties. The authors provided a comparison taking into account other literature lead-based materials where these parameters were investigated [22,33,34], from which it can be observed that the higher values of σ^2^ correspond to the higher PL quantum yields, in agreement with what was already reported by Lu and coworkers [35] for the 3-D systems MA_1−x_Cs_x_GeI_3_ and FAGeI_3_ (MA^+^ = methylammonium, FA^+^ = formamidinium). Noteworthy, Lu et al. investigated the role of pressure on the octahedral distortion of MAGeI_3_ and FAGeI_3_, achieving a similar effect as well by gradually substituting MA^+^ with the smaller Cs^+^ cation, reporting that the highest PL performance can be obtained on these perovskites by tuning the *D* value towards 0.2.

In the field of 2D perovskites, Sun and co-workers, in 2020, investigated (*R/S*-MPEA)_2_CuCl_4_, where distortions of the octahedra are caused by the Jahn-Teller effect [26]. In this paper, the authors noticed that each [CuCl_6_]^4−^ octahedron is prolonged on the Jahn-Teller *z*-axis in the CuCl plane. In HOIP systems, the magnetic spin is ascribable to the unoccupied Cu *d*_x2−y2_ orbitals [36], which are orthogonal for neighbouring octahedra in the a−b basal plane. Jahn-Teller-active ions generate cooperative anti-ferrodistortive arrangements of the neighbouring orthogonal octahedra, thus producing ferromagnetic interactions. In the same year, Jana and co-workers investigated the impact of the crystal structure on the chirality transfer mechanism and its relationship with optical properties such as CD, CPL and RD splitting [37]. For this purpose, they employed (*R/S/rac*-NPB)_2_PbBr_4_ and compared it to the already known S-MBPI [10,23]. (*R/S*-NPB)_2_PbBr_4_ displays a consistent distortion of the metal-halide octahedra, quantified through D and σ^2^, and associated with H-bonding interactions or helical distortions, while *S*-MBPI features nearly flat perovskite layers. Based on crystallographic indications on these two compounds, the authors reported that the chirality transfer not only needs the employment of a chiral spacer but also requires significant H-bonding interactions coupling the organic and inorganic sublattices, which determines the level of octahedra distortions [37]. CD measurements yielded good results for both HOIPs, suggesting the independence of this optical property from the chirality transfer. On the other hand, the CPL response unveils a decrease in the intensity ratio among the narrow free-excitonic emission and the broad self-trapped excitonic emission with the increase of structural distortions, in line with what already reported for <100>-oriented lead bromide HOIPs [38]. In addition, by performing theoretical calculations, the author ascribed the high value of RD splitting in (*R/S*-NPB)_2_PbBr_4_ to the octahedral tilting distortions and the broad distribution of Pb-Br-Pb bond angles, in agreement with the much lower value found in *S*-MBPI and consistent with simulations performed with models displaying distorted or undistorted octahedra.

In 2021, the same research group investigated several already known or *ad-hoc* synthesized HOIPs, crystallizing in non-centrosymmetric or chiral space groups, to shed more light on the parameters affecting RD splitting [29]. The authors specified that inserting a chiral cation is not sufficient to induce chirality in the inorganic layers since, to achieve a detectable inversion asymmetry, the chiral cation needs to induce distortions within the inorganic layers. For this purpose, they proposed a descriptor, Δ*β*, indicative of the difference between adjacent octahedral bond angles and studied its correlation with the computed RD spin-orbit coupling (SOC) values. As a general trend, high Δ*β* values were found in Br- and Cl-based HOIPs, while lower values were detected in I-based perovskites. This trend led to high RD SOC splitting in the former cases while absent or negligible ones in the latter cases, as disclosed by investigating the band splitting through density functional theory (DFT) calculations. Upon decomposing Δ*β* into in-plane (Δ*β_in_*) and out-of-plane (Δ*β_ou_*_t_) contributions, it was established that Δ*β_in_* best correlates with the RD splitting [29]. Notably, fitting the strong correlation of spin-splitting parameters with Δ*β_in_* results in a good overlap with the confidence intervals, implying that this empirical correlation is quantitative, at least in the considered lead-based HOIPs [29].

Again, in 2021 Lin et al. investigated the role of the para-substituent in a series of (XMBA)_2_PbI_4_ (X = H, F, Cl, Br, I). The authors reported that while the substituents influence the *d*-spacing of the HOIPs films, with the trend ((MBA)_2_PbI_4_) < ((FMBA)_2_PbI_4_) < ((ClMBA)_2_PbI_4_) < ((BrMBA)_2_PbI_4_) < ((IMBA)_2_PbI_4_), it does not play a role in the octahedral deformation, as witnessed by the comparable band gaps. From CD measurements, the authors observed that the rotatory strength, calculated in terms of *g* value, decreases as the *d*-spacing increases. As it was observed that all the considered HOIPs displayed similar absorption and emission profiles, the different *g* values should arise from the magnetic transition dipole moment term, which decreases as the *d*-spacing increases. Moreover, by performing SC-XRD measurements on (F-MBA)_2_PbI_4_ and (Cl-MBA)_2_PbI_4_ the authors unveiled the additional role of the halogen-halogen interactions in the *g* value, which increases for heavier halogens in the para position. In short, the interplay of *d*-spacing and halogen–halogen interactions led to optimal CD and CPL responses for (ClMBA)_2_PbI_4_, yielding a trend in the circular dichroism intensity, of the title HOIPs, in the order (ClMBA)_2_PbI_4_ > (BrMBA)_2_PbI_4_ > (IMBA)_2_PbI_4_ > (MBA)_2_PbI_4_ > (FMBA)_2_PbI_4_.

## 4. Concluding Remarks and Future Perspective

This Review aims to provide a landscape of the current progress in chiral HOIPs, focusing on the synthesis, the crystal structure characterization, and the investigation of the chiroptical properties with particular emphasis on lead-free materials. This is an important aspect for a developing research field that should immediately deal with the toxicity issue of Pb. As it has been shown, other less harmful metals, such as Sn, Ag, Bi, or Cu, can be used to design chiral perovskites with suitable and highly tunable properties. In the selected works, the crystal structure was characterized, mainly by SC-XRD, providing a powerful tool for evaluating the structural origin of the chiroptical properties and chirality transfer mechanism, paving the way for breakthrough advances in the design of HOIPs materials. Particular attention was focused on those works trying to set up the first structure-property correlation schemes in chiral perovskites. Several parameters related to the octahedra distortion have been proposed as potential candidates to unveil and correlate the role of long- and short-range structural distortions with the extent of chiroptical response. 

With respect to non-chiral HOIPs, the lack of a wider family of well-characterized phases, both in terms of crystal structure and dissymmetry factors, still limits the ability to establish a solid route to wise materials engineering. The field of chiral perovskite will benefit by more rigorous and systematic studies on rational series of compositions to unveil the role of structural dimensionality, i.e., moving from 2D to 1D and 0D, as well as of central metal, on the chiroptical properties. In addition, efforts should be put into designing and synthesizing novel chiral ligands (today, essentially, only those commercially available are used) to provide a more rigorous correlation between the chemical nature of the ligand and the chirality transfer mechanism.

With the future ability to devise and prepare *ad hoc* chiral perovskites, we expect that this research field will become the next “big one” in the current research for optoelectronics and, more importantly, spintronics applications.

## Data Availability

Not applicable.
